# COVID-19 Vaccination Among Students in Tunisia: Perceptions and Attitudes

**DOI:** 10.1192/j.eurpsy.2025.1139

**Published:** 2025-08-26

**Authors:** A. Guedria, Y. Akkari, R. Ayoub, S. Nabli, H. Ben Abid

**Affiliations:** 1 Child and Adolescent Psychiatry Departement, Fattouma Bourguiba Hospital; 2 University of Monastir, Monastir, Tunisia

## Abstract

**Introduction:**

COVID-19 has had a major impact on public health globally, prompting mass vaccination campaigns to curb virus transmission and severe disease outcomes. Vaccination uptake among young adults, including university students, plays a crucial role in achieving herd immunity. However, perceptions about vaccine safety, effectiveness, and necessity influence vaccination rates and acceptance.

**Objectives:**

Assessing the prevalence of COVID-19 vaccination among students at the Higher Institute of Technological Studies in Ksar Hlel, Tunisia, and exploring their perceptions regarding vaccination, including willingness to receive future doses and attitudes toward vaccine mandates.

**Methods:**

This is a cross-sectional descriptive study conducted from the 6^th^ to the 15^th^ December 2021, involving a sample of students from the Higher Institute of Technological Studies (ISET) in Ksar Helal, Tunisia, using a pre-established questionnaire. Data entry and statistical analysis were performed using SPSS software, version 22.

**Results:**

Our sample consists of 315 students, of whom 43.5% were aged between 18 and 20, and 42.9% were in their first year. The sex ratio was 0.53. Ninety-nine students (31.4%) had contracted COVID-19, with 76 rapid or PCR tests (24.1%) conducted. COVID-19 had affected 190 of the students’ family members (60.3%). COVID-19 vaccination coverage among the students was 76.8% (242 students). Among the vaccinated, 135 students (55.8%) received one dose, and 107 (44.2%) received two doses. Regarding the reasons for vaccination, 183 students (75.6%) accepted the vaccine to prevent transmitting COVID-19 to their family or friends, 179 students (74%) to protect themselves from the virus, and 152 (62.8%) to help end the COVID-19 pandemic. Adverse effects from vaccination were reported by 86% of students (208) including headaches (97.9%), arm pain (68.4%), and fever (58.3%). In our sample, 202 students (64.1%) were convinced of the benefits of vaccination, while 176 (55.9%) opposed mandatory vaccination, and 228 (72.4%) were against receiving a third dose. Among the unvaccinated students, 11 (15.1%) expressed confidence in the vaccination and indicated willingness to get vaccinated in the future.

**Conclusions:**

The study reveals a high prevalence of COVID-19 vaccination among students, with a majority expressing confidence in the vaccine’s benefits. However, there is notable resistance to mandatory vaccination and additional doses, indicating mixed perceptions and lingering concerns.

**Disclosure of Interest:**

None Declared

